# Editorial: Nutraceuticals and functional foods in chronic disease prevention and treatment

**DOI:** 10.3389/fnut.2025.1706190

**Published:** 2025-10-20

**Authors:** Alina Kurylowicz

**Affiliations:** ^1^Department of Human Eigenetics, Mossakowski Medical Research Centre, Polish Academy of Sciences, Warsaw, Poland; ^2^Department of Internal Medicine and Geriatric Cardiology, Centre of Postgraduate Medical Education, Warsaw, Poland

**Keywords:** nutraceuticals, functional foods, polyphenols, dietary fiber, trehalose, D-psicose, prebiotics

In recent years, our understanding of the role nutraceuticals and functional foods play in the prevention and treatment of chronic diseases has increased significantly. By providing bioactive compounds and essential nutrients, nutraceuticals and functional foods modulate inflammation, improve metabolism, enhance immune function, and reduce risk factors associated with these diseases. This Research Topic of Frontiers in Nutrition collects original articles and meta-analyses on interventions using various natural and functional foods or compounds related to cardiovascular health, metabolic disorders, and associated conditions.

Cardiovascular diseases are currently the leading cause of death worldwide, and their prevention is a key element of health promotion strategies. In this Research Topic, Jia et al.'s meta-analysis clearly shows the beneficial effects of consuming polyphenol-rich seeds (e.g., Brazil nuts, almonds, and flaxseed) on lipid profiles and inflammatory parameters in patients with coronary heart disease (CHD). In turn, a meta-analysis by Arjmandfard et al. summarizes recent studies on the effects of apple cider vinegar on glycemic control and insulin sensitivity in patients with type 2 diabetes, showing that this intervention reduces fasting glucose and HbA1c levels and improves insulin secretion in a dose-dependent manner. A series of meta-analyses, conducted by Jangid et al., complements a study covering papers published over the past 60 years and highlights the potential of cranberry-derived bioactive compounds, particularly proanthocyanidins (PACs), in preventing and treatintg urinary tract infections.

The 21st century has brought about a pandemic of obesity and related complications; in addition to the meta-analyses mentioned above, the Research Topic includes several original papers evaluating the potential of nutritional interventions for various metabolic disorders. Liu et al. demonstrate the beneficial effect of black chokeberry (Aronia melanocarpa) on oxonic acid-induced hyperuricemia in mice, comparable in efficacy to allopurinol. Two studies on a mouse model with a high-fat diet (HFD) suggest the potential of Polygonatum sibiricum insoluble dietary fiber (PIDF) and the natural disaccharide trehalose to reduce hyperlipidemia, body weight, and improve carbohydrate metabolism (Ma et al.; Yeh et al.). As one of the consequences of the obesity pandemic is an increasing frequency of steatotic liver disease, this Research Topic includes two original research studies by Tan et al. and Tang et al., who conducted experimental studies on the use of nutraceuticals in treating liver diseases. The first study utilized a mouse model of liver steatosis to demonstrate the potential of D-psicose (DPS), a sucrose substitute providing only 0.3% of sucrose's energy content, in reducing lipid accumulation, inflammation, and oxidative stress parameters in this organ. The second study found that the traditional Chinese herbal formula, Liuweizhiji Gegen-Sangshen Beverage (LGS), activates the SCFAs/GPR43/GLP-1 pathway, reducing liver damage in Alcoholic Liver Disease (ALD). Notably, both nutraceuticals exerted a beneficial effect on the microbiome. Subsequently, three consecutive articles in this Research Topic are devoted to dietary interventions that modulate gut microbiota. A study by Lian et al. demonstrates the beneficial, synergistic effect of cistanche polysaccharides and acteoside in regulating gut microbiota diversity and increasing the number of beneficial bacteria in rats. In turn, Wei et al. show in their work that restoring normal gut microbiota can be achieved through the use of a hawthorn postbiotic probiotic, which regulates intestinal water and sodium metabolism, maintains the intestinal barrier, promotes epithelial cell proliferation, reduces inflammatory responses, and improves short-chain fatty acid metabolism. Finally, the work by Bellomo et al. provides evidence of the potential of milk-based postbiotics from Lactobacillus plantarum to reduce gliadin peptide-induced inflammation *in vitro* and in intestinal organoids from patients with celiac disease.

We hope that these papers in this Research Topic will inspire future research on nutraceuticals and functional foods for the prevention and treatment of chronic diseases.

